# Tracing the origin of *Treponema pallidum* in China using next-generation sequencing

**DOI:** 10.18632/oncotarget.10154

**Published:** 2016-06-17

**Authors:** Jun Sun, Zhefeng Meng, Kaiqi Wu, Biao Liu, Sufang Zhang, Yudan Liu, Yuezhu Wang, Huajun Zheng, Jian Huang, Pingyu Zhou

**Affiliations:** ^1^ STD Institute, Shanghai Skin Disease Hospital, Shanghai, China; ^2^ Oncology Bioinformatics Center, Minhang Hospital, Fudan University, Shanghai, China; ^3^ School of Laboratory Medicine, Wenzhou Medical University, Wenzhou, Zhejiang, China; ^4^ Shanghai Skin Disease Hospital, Clinical School of Anhui Medical University, Shanghai, China; ^5^ Shanghai-MOST Key Laboratory for Disease and Health Genomics, Chinese National Human Genome Center and National Engineering Center for Biochip at Shanghai, Shanghai, China; ^6^ Key Laboratory of Systems Biomedicine (Ministry of Education) and Collaborative Innovation Center of Systems Biomedicine, Shanghai Center for Systems Biomedicine, Shanghai Jiao Tong University, Shanghai, China

**Keywords:** Treponema pallidum, syphilis, NGS, SNVs, evolution, Pathology Section

## Abstract

Syphilis is a systemic sexually transmitted disease caused by *Treponema pallidum* ssp*. pallidum* (TPA). The origin and genetic background of Chinese TPA strains remain unclear. We identified a total of 329 single-nucleotide variants (SNVs) in eight Chinese TPA strains using next-generation sequencing. All of the TPA strains were clustered into three lineages, and Chinese TPA strains were grouped in Lineage 2 based on phylogenetic analysis. The phylogeographical data showed that TPA strains originated earlier than did *T. pallidum* ssp*. pertenue* (TPE) and *T. pallidum* ssp*. endemicum* (TPN) strains and that Chinese TPA strains might be derived from recombination between Lineage 1 and Lineage 3. Moreover, we found through a homology modeling analysis that a nonsynonymous substitution (I415F) in the PBP3 protein might affect the structural flexibility of PBP3 and the binding constant for substrates based on its possible association with penicillin resistance in *T. pallidum*. Our findings provide new insight into the molecular foundation of the evolutionary origin of TPA and support the development of novel diagnostic/therapeutic technology for syphilis.

## INTRODUCTION

*Treponema pallidum* ssp*. pallidum* (*T. pallidum* ssp*. pallidum*; TPA) is the causative agent of syphilis [1] and infects more than 12 million people worldwide annually [2–4]. In China, although the elimination of syphilis was announced in the 1960s [5], the incidence of the disease has increased rapidly in recent years [6, 7]. The number of reported syphilis cases increased more than 50-fold within 12 years, from 1993 to 2005 [8], and the latest Chinese national surveillance data of incidence rates for 2014 and 2013 were 419,091 and 406,772 cases, respectively (http://english.nhfpc.gov.cn/). Because syphilis cases were reported as early as the 16^th^ century in China [5], it is necessary to investigate the origin of the currently circulating TPA strains and to determine the transmission linkage between syphilis and global TPA strains.

Until recently, the genotyping of TPA strains relied on the characterization of the acidic repeat protein gene (*arp*), the *tp0548* gene and the restriction fragment length polymorphism (RFLP) analysis of *Treponema pallidum* repeat gene (*tpr*) subfamily II (*tpr*E, *tpr*G, and *tpr*J), which was used to infer the prevalence and geographical distribution of TPA strains worldwide, the relationship between strain types and the prevalence of neurosyphilis [9, 10]. However, the *tpr* gene family also displays wide genetic diversity among various subspecies of *T*. *pallidum* (syphilitic vs. non-syphilitic), supporting its critical roles in understanding bacterial evolution and immune-escaping mechanisms and in developing a vaccine against pathogenic treponemes [11]. To date, most TPA genomic sequence data are derived from American TPA strains. However, the genetic base underlying the evolution and emergence of Chinese TPA strains remain unclear.

As in many bacteria [12], whole-genome sequencing and phylogenetic linkages remain preferable for demonstrating the genetic relationships and molecular epidemiological characteristics of *T. pallidum*. Thus far, 12 full-genome sequences of treponemes have been published, including those of the TPA Nichols strain (GenBank: AE000520.1 and CP004010.2) [13, 14], TPA DAL-1 strain (GenBank: CP003115.1) [15], TPA Mexico A strain (GenBank: CP003064.1) [16], TPA Sea81-4 strain (GenBank: CP003679.1) [17], TPA SS14 strain (GenBank: CP004011.1 and CP000805.1) [13, 18], TPA Chicago strain (GenBank: CP001752.1) [19], *T. pallidum* ssp*. pertenue* (TPE) Samoa D strain (GenBank: CP002374.1) [20], TPE CDC-2 strain (GenBank: CP002375.1) [20], TPE Gauthier strain (GenBank: CP002376.1) [20], TPE Fribourg-Blanc strain (GenBank: CP003902.1) [21], *T. pallidum* ssp*. endemicum* (TPN) Bosnia A strain (GenBank: CP007548.1) [22], and *T. paraluiscuniculi* strain Cuniculi A (a rabbit treponemal isolate, GenBank: CP002103.1) [23]. These strains have contributed to the current understanding of the genetic features of treponemal species. As previously described [24–26], the members of the genus *Treponema* have high genetic similarity. Despite previous studies focusing on genetic variations in TPA or TPE strains or other treponemes [20–22, 27–29], there is little evidence regarding the origin and dynamic evolution of global treponemal species. Notably, the theory of an Old World origin of venereal syphilis suggests that TPA originated in Europe [30, 31], while the New World hypothesis posits that TPA was introduced from America to Europe [32, 33]; thus, the origin or ancestry of TPA strains remains controversial and contradictory.

In the present study, we analyzed the genome sequences of TPA strains using next-generation sequencing technology to precisely define the genetic differences and origin of TPA in China.

## RESULTS

### Sequenced genomes of eight Chinese TPA isolates

To investigate the genetic variations present in TPA strains in China, eight DNA samples extracted from TPA-infected rabbit testes were sequenced using next-generation sequencing. A total of 20,869,470-37,469,842 high-quality reads per sample were determined (Table 1, Supplementary Table 1). Of the unique reads, 95.61% could be mapped to the reference genome (Nichols strain, GenBank: CP004010.2), and 99.99% of the TPA genomic sequences were covered at a depth of at least 55-fold in each sample (Table 1). The mean depth of coverage was approximately 1014-fold, and the greatest depth of coverage was 2253-fold in the SHD-R sample (Table 1). The lowest depth of coverage was 55-fold in the B3 sample, which may be associated with the lower number of treponemal cells in this sample (Table 1).

**Table 1:** Background information and sequencing statistics for genome of *T. pallidum*

Strain	Origin Place	Year of isolation	Raw data	Clean data	Ratio	unique reads	unique ratio(%)	Coverage (%)	Mean depth	SNV number	Count of treponemes
SHC-0	Shanghai	2014	40416294	37469842	92.71%	33483604	89.36%	99.99%	1899.73	286	1×10^7^
SHD-R	Shanghai	2014	28323906	26245436	92.66%	21129166	80.51%	99.99%	2253.37	282	1×10^7^
SHE-V	Shanghai	2014	26352892	24296460	92.20%	23352990	96.12%	99.99%	413.53	277	1×10^7^
SHG-I2	Shanghai	2014	23141660	20869470	90.18%	20312002	97.33%	99.99%	163.98	279	1×10^7^
B3	Shanghai	2015	26527340	26340014	99.29%	26046390	98.89%	99.99%	55.28	243	1×10^6^
C3	Shanghai	2015	26746096	26581754	99.39%	25883586	97.37%	99.99%	487.29	268	7×10^7^
K3	Shanxi	2015	25504866	25293142	99.17%	24091738	95.25%	99.99%	796.43	284	1×10^7^
Q3	Anhui	2015	24819110	24649998	99.32%	20028148	81.25%	99.99%	2047.53	281	1×10^7^

### Genomic diversity of TPA strains in China and other countries

**Figure 1:**
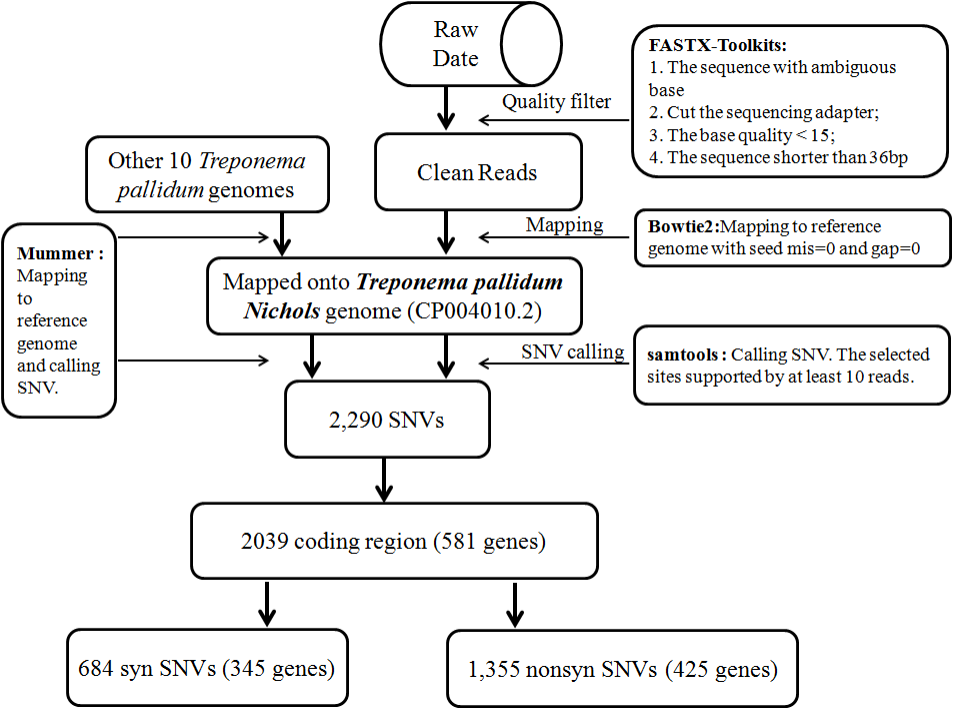
Flowchart for analysis of the data derived from next-generation sequencing

To clarify the genetic diversity of *Treponema*, we obtained 11 treponemal genomic sequences from GenBank to analyze SNVs, including 6 TPA strains: Nichols, Chicago, Mexico A, SS14, DAL-1 and Sea81-4; 4 TPE strains: Gauthier, Samoa D, CDC2, and Fribourg-Blanc; and 1 TPN strain: Bosnia A. All of the genomic sequences were matched to the Nichols strain genome using Bowtie2, and the SNVs were subsequently annotated using SAMTools (Figure 1).

Treponemal species shared high similarity during their genetic evolution (genomic identity >99.5%) [20, 25, 27]. The number of SNVs found in individual Chinese strains ranged from 243 to 286 (median 280) and displayed >70% similarity among the isolates. Fewer SNVs were identified in the genomes of the Chicago and DAL-1 strains compared to the Nichols strain (median 17 and 34, respectively). In contrast, the total number of SNVs in the genomes of the SS14, Mexico A and Sea 81-4 strains ranged from 299 to 397 (median 324), representing the maximum number of SNVs compared to the Nichols strain among TPA subspecies. Furthermore, the number of SNVs in the TPE and TPN strains ranged from 1220 to 1467 (medium 1267), which was significantly higher than that in the TPA strains (Supplementary Table 2). The distinct pattern of SNVs identified among various TPA strains suggested distinct genetic relationships between these TPA strains. Therefore, we subsequently performed a genomic SNV-based phylogenetic analysis to determine the genetic linkage of these TPA strains.

**Figure 2:**
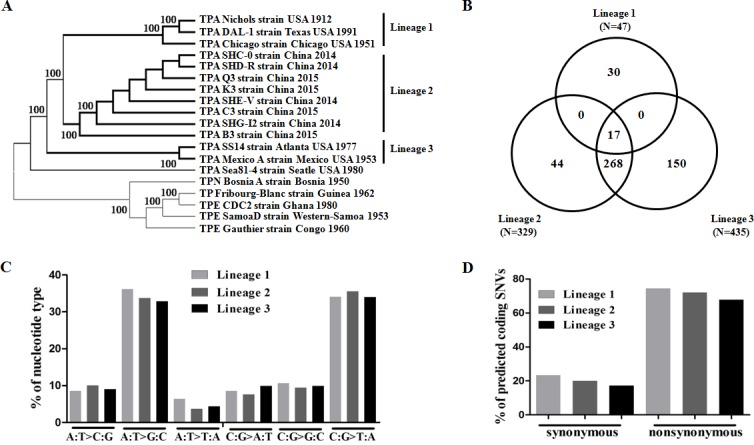
Characteristics of SNVs in different TPA lineages **A.** Neighbor-joining phylogeny analysis of 697 SNVs defining TPA lineages. **B.** Venn diagram showing the number of SNVs in different TPA lineages. **C.** Percentage of mutation spectra of SNVs in different TPA lineages. **D.** Percentage of nonsynonymous and synonymous SNVs in different lineages.

As expected, all 14 of the TPA strains formed a large clade with a 100% bootstrap value in the maximum-likelihood tree, whereas five TPE and TPN strains formed another clade (Figure 2A, Supplementary Figure 1), suggesting that both of these groups of strains formed independent subspecies during evolution. Furthermore, three distinct clusters were observed within the TPA clade: Cluster 1 (Nichols, DAL-1, and Chicago), Cluster 2 (Chinese TPA, SHC-0, SHD-R, SHE-V, SHG-I2, B3, C3, K3, and Q3) and Cluster 3 (Mexico A and SS14). Strain Sea81-4 was separated from these strains and was positioned more closely to Cluster 3. Consistent with the observed SNV pattern among various treponemal species, the strains with high-similarity SNV sites were clustered together. These results suggest that multiple independent lineages may have formed during the long-term evolution of TPA strains. To confirm this possibility, a Bayesian inferred tree of genomic SNVs was reconstructed using MrBayes 3.1 as previously described [34], and the results revealed the same phylogenetic relationships as did the ML tree (data not shown). Therefore, the three independent clusters were designated as three TPA lineages: Lineage 1 (Nichols, DAL-1, and Chicago), Lineage 2 (SHC-0, SHD-R, SHE-V, SHG-I2, B3, C3, K3, and Q3), and Lineage 3 (Mexico A and SS14). In particular, Lineage 1 was closer to Lineage 2 than to Lineage 3 within the TPA strains in the genomic SNV-based ML tree (Figure 2A).

To investigate SNVs in various TPA lineages using a Venn diagram (Figure 2B), a total of 47, 329 and 435 SNVs were identified in the genomes from Lineage 1, Lineage 2, and Lineage 3, respectively. Lineage 1 did not share any common SNVs with Lineage 2 or with Lineage 3, whereas Lineage 2 and Lineage 3 shared 268 SNVs. Notably, more SNVs were shared between Lineage 2 and Lineage 3 than between Lineage 2 and Lineage 1. However, Lineage 2 showed a closer genetic linkage to Lineage 1 than to Lineage 3, as indicated by the genomic SNV-based ML tree (Figure 2A). Although further genetic analyses are required to interpret this phenomenon, it is possible that Lineage 3 shares some identity with treponemal strains in addition to TPA (e.g., TPE or TEN strains). Furthermore, a total of 224 SNVs were designated as lineage-specific SNVs—30 in Lineage 1, 44 in Lineage 2 and 150 in Lineage 3 (Figure 2B, Supplementary Table 3)—which were mainly distributed in the *tpr* gene family and encoded for hypothetical proteins or methyl-accepting chemotaxis proteins (mcp2-1) (Table 2). The *tpr* family genes experienced immune pressure *in vivo* and were employed for the evolutionary analysis and subtyping of TPA strains [35–37]. The distribution of lineage-specific SNVs also confirmed the critical role of *tpr* family genes in the genetic evolution of TPA strains. In particular, Lineage 3 exhibited specific SNVs in genes encoding for methyl-accepting chemotaxis proteins (mcp2-1), which were previously identified from recombination events with TPE strains [16], suggesting a possible genetic linkage of the Mexico A strain with TPE strains.

**Table 2:** Number of SNVs in the hree TPA lineages

	Lineage 1	Lineage 2	Lineage 3
**Tpr protein(*N*= 71)**			
tprC			11
tprD			20
tprI			4
tprE		3	
tpr G		1	
tpr K	4	22	
tpr L			6
**Hypothetical protein and putative outer membrane protein (*N*= 50)**			
TPANIC_0136	18		9
TPANIC_0548			13
Hypothetical protein	1	1	8
**Intergenic (*N*= 49)**		5	44
**Other function(*N*= 54)**			
Penicillin-binding protein		4	
Methyl-accepting chemotaxis protein (mcp2-1)			24
others	7	8	11
**Total**	30	44	150

Among the SNVs that were characterized in the three lineages, there was an obvious tendency of nucleotide mutations toward C:G>T:A and A:T>G:C in all of the lineages (Figure 2C). Notably, the C:G>G:C transversion was also significantly enriched in Lineage 1. Regarding the enrichment of either the A:T>G:C or C:G>T:A mutation, the GC content (53%) of the genomes was not altered in all of the lineages, indicating that the biases of these transversions might not affect the usage of the codons and amino acids in TPA strains grown under different selective pressures [38, 39]. In addition, a higher proportion of nonsynonymous SNVs was displayed in all of the lineages (Figure 2D), potentially affecting the function of the respective gene-encoded products. To investigate these effects, we focused our analyses on nonsynonymous SNVs and found that 11 genes (≥2 SNVs per gene) were most highly affected, some of which were potential membrane genes (*TPANIC_0136; TPANIC_0326; TPANIC_0548; TPANIC_0865* and *TPANIC_0515*) or known virulence factors, such as *tpr* family genes (*TPANIC_0897*; *TPANIC_0317*; *TPANIC_0313* and *TPANIC_1031*) and penicillin-binding protein (*TPANIC_0760*). Although the most highly affected genes were non-specific for unique lineages, Lineage 2 contained the maximum number of *tpr* genes that were highly affected by SNVs. Given that the reference strain spread among European and US populations, these lineage-specific SNVs found in *tpr* family genes in the Chinese strains might reflect selective pressure in Chinese or Asian populations.

### Potential effects of SNVs on the response of syphilis patients to penicillin treatment

**Figure 3:**
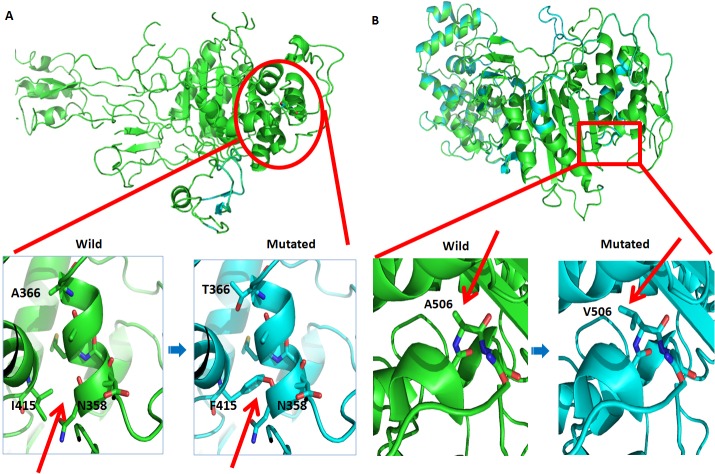
Predication of SNV effects on PBP3 and PBP2 proteins by homology model analysis **A.** Ribbon representation of the PBP3 protein as predicted by the Phyre2 Server. Red arrows show the structural alteration caused by the SNVs compared to the wild-type model. **B.** Ribbon representation of the PBP2 protein as predicted by the Phyre2 Server. Red arrows show the structural alteration caused by the SNVs compared to the wild-type model.

Penicillin has been recommended worldwide as a first-line drug for the treatment of all stages of syphilis [2]. Although there is no evidence available for penicillin resistance in *T. pallidum*, penicillin treatment failure has caused concern [40–44]. In this study, we identified seven SNVs in penicillin-regulatory genes, including five nonsynonymous mutations in penicillin-binding proteins (PBPs) and two synonymous mutations in a penicillin-tolerance protein (lytB) (Table 3). Because a nucleotide mutation in endogenous PBPs might lead to resistance against β-lactams [11,45], we subsequently predicted the effects of five nonsynonymous SNVs on the function of PBP genes in Lineage 2 and Lineage 3. Two nonsynonymous SNVs (G773571A, A506V, in PBP2 protein and A825069T, I415F, in PBP3 protein) in Chinese TPA strains were likely to impair the function of proteins as predicted through a SIFT analysis (Table 3). A homology modeling analysis revealed that the I415 to F415 substitution altered the structure of side chains, which affected the structural flexibility of the PBP3 protein and might thereby change the binding constant for substrates of some proteins (Figure 3A). In contrast, the A506 to V506 mutation did not cause any structural alterations, although a deleterious effect was indicated in the SIFT analysis (Table 3, Figure 3B). These results suggest that the SNVs present in PBP genes might compromise the responses of syphilis patients to penicillin treatment, although more evidence is needed to confirm these results in the future.

**Table 3:** The SNVs of penicillin regulatory Proteins in all Lineages

Gene	Annotation	SNV	Mutation	Mutation type	Lineage	Strain	SIFT Score^a^
*TPANIC_0500*	penicillin-binding protein (pbp-1)	C537571T	P564L	nonsynonymous	2,3	Strain C-0, D-R, E-V, G-I2, B3, C3, K3, Q3 and SS14	0.2
*TPANIC_0547*	4-hydroxy-3-methylbut-2-enyl diphosphate reductase/penicillin tolerance protein (lytB)	T592290C	/	synonymous	2,3	Strain C-0, D-R, E-V, G-I2, B3, C3, K3, Q3, SS14 and Mexico A	/
		T592614C	/	synonymous	2,3	Strain C-0, D-R, E-V, G-I2, B3, C3, K3, Q3, SS14 and Mexico A	/
*TPANIC_0705*	penicillin-binding protein (pbp-2)	G773571A	A506V	nonsynonymous	2	Strain C-0, D-R, E-V, G-I2, B3, C3, K3 and Q3	0.03
*TPANIC_0760*	penicillin-binding protein (pbp-3)	G824922A	A366T	nonsynonymous	2	Strain C-0, and B3	0.14
		A825069T	I415F	nonsynonymous	2	Strain C-0, D-R, E-V, G-I2, B3, C3 and Q3	0.03
		C825071G	I415M	nonsynonymous	2	Strain K3	0.17

a functional SNVs predicted using SIFT (SIFT score ≤0.05 as the deleterious effect).

### The SNVs validated by Sanger sequencing

To validate the variants identified by NGS, we amplified and sequenced 13 genes and 3 intergenic regions covering 23 SNV sites, including 6 SNVs in the PBPs gene, 7 SNVs in the *tpr* genes and 10 randomly selected SNVs, using PCR-based Sanger sequencing technology (ABI 3730 DNA Analyzer, Applied Biosystems). The PCR primers are shown in Supplementary Table 4. The results confirmed 23 (100%) of the 23 candidate SNVs (Supplementary Figure 2), suggesting that NGS is a highly sensitive and reliable method for identifying SNVs.

### Tracing the origin of Chinese TPA strains

As most of the identified lineage-specific SNVs occurred in the *tpr* gene family, the *tpr* genes were used to further trace the origin of Chinese TPA strains. The *tpr* gene family comprises 3 gene subfamilies and 12 paralogous genes: Subfamily I (*tpr*C, D, I and F), Subfamily II (*tpr*E, G, and J), and Subfamily III (*tpr*A, B, H, K, and L). As potential outer membrane proteins and important virulence factors, the gene products from Subfamilies I and II exhibited conserved amino and carboxyl terminal regions and variable central regions, whereas Subfamily III presented highly variable regions across the full length of the genes [46, 47]. Due to the genetic characteristics of the various subfamilies of genes, both previous reports and the results of the present work indicated that the members of Subfamilies I and II are more suitable for the analysis of genetic evolution and might form the same species-specific cluster in a phylogenetic tree as did genomic SNVs (Supplementary Figures 3 and 4), whereas the Subfamily III members never cluster in a species-specific manner [11].

**Figure 4:**
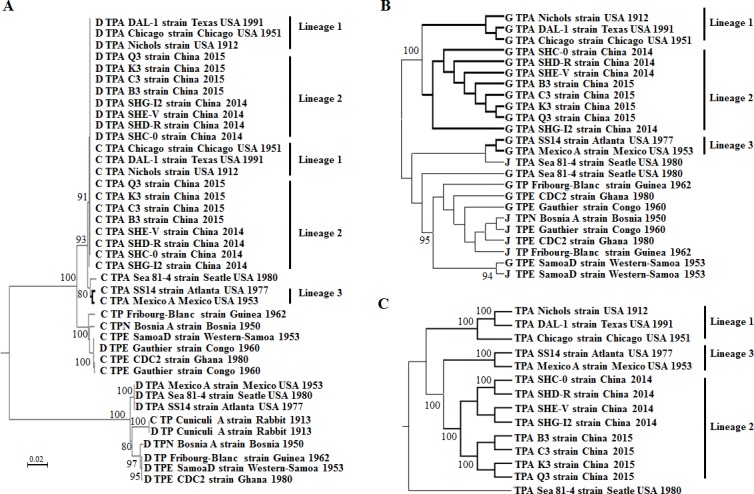
Phylogenetic analysis based on the *tpr* genes and SNVs of *T. pallidum* . **A.** Phylogenetic tree showing clusters of Subfamily I of *tpr* genes. **B.** Phylogenetic tree showing clusters of Subfamily II of *tpr* genes. **C.** Phylogenetic tree showing clusters of *tpr* genes based on genomic SNVs after the removal of SNVs from the *tprD* and *tprG* genes. The tree was constructed using MrBayes 3.1. Significant linkages using Bayesian phylogenetic inference analysis were considered as probabilities of 100%.

In the phylogenetic analysis of Subfamily I genes, three clades were presented with a 99% bootstrap value: Clade I included all of the *tpr*C genes and some of the *tpr*D genes (mainly from TPA Lineages 1 and 2); Clade II included the *tpr*D genes from TPA strains (SS14, Mexico A and Sea 81-4) and TPE strains; and Clade III included the *tpr*F and *tpr*I genes (Supplementary Figure 3). There were three clusters in Clade I: all of the *tpr*C and *tpr*D genes from TPA Lineage 1 and Lineage 2 strains formed Cluster I with a 91% bootstrap value, while those from the Lineage 3 strains and Sea81-4 formed Cluster II with an 80% bootstrap value. These two clusters formed one large TPA cluster with a 93% bootstrap value. Outside this TPA cluster, the *tpr*C genes from TPE and TEN strains formed Cluster III with a 100% bootstrap value, suggesting that the *tpr*C genes of Chinese TPA strains are most likely derived from Lineage 1 rather than Lineage 3, although the latter also showed a close relationship with the *tpr*C gene cluster of Lineage 1 and Lineage 2 strains (Figure 4A). Furthermore, the findings suggested that all of the *tpr*C genes of treponemal species had the same origin and that gene conversion from *tpr*C to *tpr*D might have occurred in TPA Lineage 1 and 2 strains. Only the *tpr*D genes derived from Lineage 3 and the TPE and TPN strains were included in Clade II and formed a clade with a 100% bootstrap value (Supplementary Figure 3, Figure 4A). In Clade III, the *tpr*F and *tpr*I genes from all of the TPA strains formed one cluster, whereas the *tpr*F and *tpr*I genes from TPE and TPN strains formed another cluster (Supplementary Figure 3).

In the phylogenetic analysis of Subfamily II genes, there were four clusters with high bootstrap values: all of the *tpr*G genes and TPE and TPN-derived *tpr*J genes formed Cluster I; the *T. paraluiscuniculi* strain-derived *tpr*E, *tpr*G and *tpr*J genes formed Cluster II; all of the TPA-derived *tpr*J genes formed independent Cluster III; and all of the *tpr*E genes derived from treponemes (except for the *T. paraluiscuniculi* strain) formed Cluster IV (Supplementary Figure 4). As shown in Cluster I (Supplementary Figure 4), the *tpr*G genes of the Chinese TPA strains were most likely derived from Lineage 1 rather than Lineage 3. Similar to the *tpr*D genes, the *tpr*G genes of Lineage 3 showed high homology with the genes of TPE and TPN strains, as shown in Cluster II (Figure 4B). Gene conversions were indicated between the *tpr*J and *tpr*G genes in the TPE and TPN strains and the TPA Sea81-4 strain in addition to the *tpr*E, *tpr*G and *tpr*J genes from *T. paraluiscuniculi* strains. Because the *tpr*G genes constituted the majority of Cluster I, *tpr*G might have been the donor gene for gene conversion. Moreover, the *tpr*E genes were highly conserved among members of the genus *Treponema*, whereas the *tpr*J genes were highly conserved in TPA strains (except in Sea81-4).

Intragenomic gene conversion might have occurred between the *tpr*C and *tpr*D genes of Lineage 2 (Chinese TPA strains) and Lineage 1 (Figure 4A) and between the *tpr*F and *tpr*I genes and *tpr*G and *tpr*J genes of TPE and TPN strains (Supplementary Figure 3), as confirmed by the results of the present study and those of previous reports [36]. Additionally, gene conversion between *tpr*C and *tpr*D genes was identified in the TPE Gauthier strain. In addition, the high homology of the *tpr*D and *tpr*G genes between TPA Lineage 3 and the TPE and TPN strains suggested a close genetic linkage between these two species at the beginning of treponemal evolution. As noted above, although many more SNVs were shared between TPA Lineage 2 and Lineage 3 than between Lineage 2 and Lineage 1 (268 vs. 0), Lineage 2 displayed a closer genetic relationship with Lineage 1 than with Lineage 3 in the genomic SNV-based ML tree, likely reflecting the close genetic relationship between Lineage 3 and the TPE and TPN strains. To address this concern, after these SNVs were removed from the *tpr*D and *tpr*G genes, the modified genomic SNVs were used to reconstruct an ML tree. As expected, three lineages were observed in the TPA strain clade, although Lineage 2 and Lineage 3 displayed a closer genetic relationship, forming one large cluster with high confidence (Figure 4C). These results suggest that Lineage 2 exhibited a mosaic genomic structure, involving the insertion of Lineage 1-derived genes (mainly *tpr* genes) into the Lineage 3-derived genomic backbone, further suggesting that Lineage 2 might have been the product of lateral gene transfer or recombination between Lineage 1 and Lineage 3.

**Figure 5:**
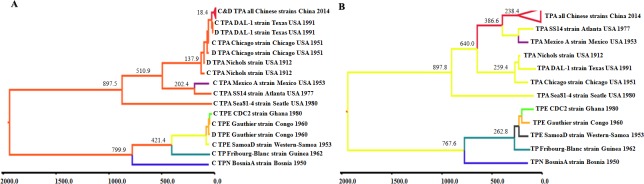
Maximum clade credibility trees of *tpr* family gene sequences and modified SNVs of *T. pallidum* Phylogeographical trees were constructed using BEAST V1.6.2. The tree branches are colored according to their respective cluster or branch. The tMRCA of each cluster is labeled at the tree nodes. **A.** Phylogeographical analysis showing the clusters based on the *tprC* and *tprD* gene sequences among treponemal strains. **B.** Phylogeographical analysis showing clusters based on the genomic SNVs of *tpr* family genes after removing the SNVs of *tprD* and *tprG* genes. tMRCA stands for time to the most recent common ancestor.

To trace the origin of Chinese TPA strains and to estimate the timeline of global TPA strains, phylogeographical analyses were performed on the *tpr*C and *tpr*D genes from Clade I of Subfamily I using the BEAST v1.7.2 software package. As indicated in Figure 5A, the TPA and TPE and TPN strains formed an independent clade during their genetic evolution, and the same three lineages were identified as in the ML tree. Notably, the tMRCA of the TPA strains occurred approximately 897 (range 465-1363) years ago, while that of the TPE and TPN strains occurred approximately 799 (range 446-1178) years ago, suggesting that the TPA strains originated earlier than did the TPE and TPN strains. The *tpr*C gene appeared earlier in evolution than did the *tpr*D gene in two independent clusters (TPA cluster and TPE and TPN cluster), suggesting that the *tpr*C gene was most likely the donor of the *tpr*D gene via gene conversion. Within the TPA clade, Sea81-4 represents the earliest TPA isolate, and the cluster formed by the three TPA lineages might represent the most recent ancestor strain for the global outbreak of TPA: the timing of this ancestor was estimated at approximately 510 years ago, near the time when syphilis infections were first reported in Europe. Three current TPA lineages appeared sequentially in the timeline: Lineage 3 first appeared approximately 202 (98-314) years ago, Lineage 1 appeared approximately 137 (67-215) years ago, and the Chinese TPA strains appeared approximately 18 (9-28) years ago.

Considering the distinct genetic linkages of Chinese TPA strains with Lineage 1 and Lineage 3 in various genomic regions, we further performed a phylogeographical analysis on modified SNV alignments (removing SNVs in *tpr*D and G genes). As expected, Lineage 2 and Lineage 3 exhibited a close relationship and shared the most recent common ancestor, whereas Lineage 2 displayed a much earlier tMRCA for modified genomic SNVs than for the *tpr*C genes (238 vs. 18.4 years ago, Figure 5B), further establishing that Lineage 2 originated from multiple distinct parental strains. Although different tMRCAs of the three TPA lineages were estimated for genomic SNVs in contrast to the *tpr*C gene, the order of the timeline of these lineages was the same in these two Markov chain Monte Carlo (MCMC) trees, with Sea81-4 representing the earliest TPA isolate and with three lineages appearing sequentially in various timelines: Lineage 3 the earliest, Lineage 1 the second earliest, and Lineage 2 the most recent. Similar to the timeline for the *tpr*C and D genes, the tMRCA for the TPA strains in the genomic SNV-based MCMC tree occurred approximately 897 years ago, while the tMRCA for the TPE and TPN strains occurred approximately 767 years ago, further confirming that the TPA strains originated earlier than did the TPE and TPN strains. Overall, both the *tpr* gene and genomic SNV-based phylogeographical analyses firmly support the distinctive evolutionary characteristics of various treponemal species and clearly estimate the genesis and tMRCAs of treponemal species.

## DISCUSSION

The first characterized genetic diversity of Chinese syphilis shows the utility of the NGS strategy for performing comparative genomic analysis of treponemal strains. To our knowledge, this is the first effort to sequence the entire TPA genome in China. Although it is widely accepted that TPA strains share high genetic homology, we revealed a distinct distribution pattern of SNVs in various TPA strains. In contrast to the Nichols strain, all of the Chinese TPA strains displayed an intermediate degree of variation (243-286 sites) in terms of genomic SNVs, while the Chicago and DAL-1 strains displayed minimal variation (<50 sites) and the Mexico A, SS14 and Sea81-4 strains displayed maximal variation (>300 sites). In this context, three independent TPA lineages were first identified through both SNV patterns and a phylogenetic analysis, and the strains within the lineage showed high similarity regarding SNV variation; e.g., the Chinese TPA strains shared more than 70% (236 of 329) of their SNVs. Notably, the majority of the SNVs identified in TPA strains consisted of nonsynonymous sites, which likely altered the encoded amino acids. Moreover, most lineage-specific SNVs occurred in *tpr* genes and some hypothetical genes. Although little is known about the function of these hypothetical proteins, the high frequency of SNVs observed in some hypothetical proteins is important for pathogenesis or immune escape, which deserves further study. Therefore, many lineage-specific SNVs not only reflect the imprint of genetic evolution but also affect the function of genes and even the biological characteristics of TPA strains.

In the Chinese TPA strains, most lineage-specific SNVs occurred in *tpr* genes and some hypothetical genes. Although little is known regarding the function of these hypothetical proteins, the high frequency of SNVs observed in some hypothetical proteins is important for pathogenesis or immune escape, which deserves further study. Tpr proteins play a critical role in the binding and recognition activity of TPA strains and are widely recognized by the host immune system [48, 49]. Thus, scientists have made great efforts in recent years to develop a *tpr-*gene-based vaccine against TPA infection [49].

Although penicillin therapeutic regimens have been highly effective for syphilis in the past half century, concerns have been raised that penicillin may be inadequate because “relapse” often occurs after penicillin therapy [40–44]. The lack of evidence to reasonably explain this treatment failure has plagued doctors for a long period of time. Nonsynonymous SNVs were first identified in the gene family of penicillin regulatory proteins in Chinese TPA strains (*TPANIC_0500, TPANIC_0760,* and *TPANIC_0705*) and Lineage 3 strains (*TPANIC_0500)*. Homology modeling and SIFT analysis revealed that only one SNV (A825069T; I415F in the PBP3 protein) had a deleterious effect on the structural flexibility and the binding constant for substrate stability, as this SNV might be associated with penicillin resistance in *T. pallidum* (Figure 3A). Our findings in this study might partially explain the doctors' concerns, although additional studies are needed to confirm this hypothesis.

The phylogenetic analysis revealed that SNVs, gene conversion and lateral gene transfer constituted the main forces driving the evolution of TPA strains. In 2006, Gray et al. investigated the evolution of the *tpr* gene among various subspecies and proposed that gene conversion has played a critical role in the evolution of *Treponema* [36]. In the present study, our phylogenetic analysis of *tpr* gene Subfamilies I and II not only confirmed this hypothesis but also further delineated the contribution of *tpr* gene conversion to the dynamic evolution of TPA strains, as is showed in *TPANIC_0136* and *TPANIC_0548*genes (Supplementary Figure 5 and 6). The intra-genomic gene conversion between *tpr*C and *tpr*D genes in TPA Lineage 1 and Lineage 2 might be responsible for the distinct evolution of Lineage 1 and Lineage 2 and might have led to the close genetic relationship between these two TPA lineages. In addition, common gene conversions between *tpr*J and *tpr*G genes in all of the TPA strains except for the Sea81-4 strain might have contributed to the development of Lineage 3, Lineage 2 and Lineage 1. In addition, common gene conversions between the *tpr*G and *tpr*J genes in the TPA Sea81-4 strain and TPE and TPN strains indicated a close genetic lineage between the TPA and TPE and TPN strains, suggesting that the Sea81-4 strain might represent the earliest TPA strain. Moreover, the close genetic relationship observed between the TPA Lineage 3 and TPE and TPN strains for the *tpr*D and *tpr*G genes also suggests that Lineage 2 was the second earliest strain. Notably, Lineage 2 showed a closer relationship with Lineage 1 than with Lineage 3 in the *tpr*C and *tpr*G gene-based phylogenetic tree, while Lineage 1 shared the maximum number of SNVs with Lineage 3. Thus, Lineage 1 was further identified as having a mosaic genomic structure, involving a Lineage-3-derived genomic backbone with the insertion of several Lineage-1-derived *tpr* genes. Collectively, the recently reemerged Chinese TPA strains might reflect gene mutations and gene recombination events between existing TPA strains. China announced the extinction of syphilis in the 1960s; however, a new outbreak of syphilis was reported in 1996. Since then, syphilis has become a major public health problem [7, 8]. Because a syphilis outbreak occurred throughout almost the entire country, tracing the origin and understanding the genetic linkage of Chinese TPA strains with global TPA strains is critical for designing rational prevention measures.

Indeed, although epidemics of syphilis or the TPA strain were observed more than 500 years ago, both the phylogenetic relationship among *Treponema* and the evolutionary history of TPA strains remain controversial. In 1961, Cockburn TA proposed that pinta was the original form, which subsequently evolved into yaws, followed by endemic syphilis and ultimately venereal syphilis [48]. However, in 1965, Hudson suggested that venereal syphilis, endemic syphilis, yaws, and pinta were not in fact distinct diseases, but rather, the same disease with various manifestations [49]. In 2005, Armelagos et al. indicated the lack of a molecular distinction among these subspecies [52]. In addition, Gray et al. suggested that the TPA strain was not later in origin than the TPE strain, whereas Harper et al. recently suggested that the TPE and TPN strains exhibited an earlier origin than that of the TPA strain [32]. A review of these studies revealed a deficiency in summarizing the dynamics of treponemal evolution and global genetic variation in the treponemal genome, which might have provided insufficient evidence and led to incomplete conclusions. In the present study, the phylogeographical analyses of both the *tpr*C and D genes and genomic SNVs clearly estimated the timeline for the genus *Treponema* and TPA strains.

During the first evolutionary phase of the genus *Treponema*, the TPA and TPE and TPN subspecies separated in genetic evolution and formed early ancestors in different timelines (879 and 799 years ago, respectively). Several scenarios have been proposed for the origin of the TPA species [31, 51]. New World versus Old World hypotheses concerning the origin of venereal syphilis has also long been debated. The results of the present study suggest that TPA and TPE and TPN strains shared a common ancestor and had the same origin. Although the Sea81-4 strain was only isolated in 1980 and showed the highest genetic homology with the TPE and TPN strains, strain Sea81-4 mostly closely resembled the earliest ancestor of TPA in genetic evolution, which not only further supports the common genesis of various treponemal species but also suggests that TPA strains originated in North America.

The global epidemic of treponemes initiated the second evolutionary phase. Similarly, the most recent common ancestor of global epidemic TPA strains occurred earlier than did that of TPE and TPN strains (510 vs. 421 years ago). The appearance of the most recent TPA ancestor suggests the acquisition of some abilities of TPA strains at this time point, which has been attributed to significant genetic variation in pathogens (such as gene conversion in the *tpr*G and *tpr*J genes). In this context, significant genetic alterations in pathogens are typically accompanied by a switch from an old host environment to a new host population, suggesting that the earliest introduction of TPA strains occurred from North America into Europe.

Considering that Europe initiated the global transmission of TPA strains during the 16^th^ century [52, 53], TPA strains subsequently evolved into two distinctive lineages. In addition, accompanied by the prevalence of colonialism, frequent global distribution might have led to multiple introductions of TPA strains to a single continent, suggesting that the common ancestor of Lineage 2 and Lineage 3 originated approximately 386 years ago and that the most common ancestor of Lineage 1 and Lineage 2 originated approximately 138 years ago. During this phase, TPA strains gradually lost the high genetic similarity of the *tpr* gene family with TPE and TPN strains.

In 1504, a Chinese doctor in Guangzhou described a syphilis-like manifestation corresponding to the earliest opening of a port to Western people [54]. The syphilis epidemic in China had been eradicated through endeavors of the Chinese government from 1958 to 1970 [5]. The re-emergence of syphilis/TPA in China in 1990 might reflect recombinant events mediated through recently introduced Lineage 1 and previously existing Lineage 3. Indeed, the phylogenetic analysis of *tpr* genes and modified SNVs provided the following evidence: 1) the Chinese TPA strains showed a closer genetic linkage of *tpr*C, *tpr*D and *tpr*G genes with Lineage 1 than with Lineage 3; 2) the Chinese TPA strains exhibited a close genetic linkage of the majority of genes with Lineage 3; and 3) gene conversions occurred in *tpr*C and *tpr*D genes from TPA Lineage 1 and Lineage 2 strains and in *tpr*G and *tpr*J genes from TPE and TPN strains. The tMRCA of Lineage 2, as estimated in the *tpr*C/D genes, occurred approximately 18 years ago, providing further evidence of the reemergence of TPA strains in China. Notably, the newly emerged TPA Lineage 2 strain will cause a new wave of global transmission, which will also require serious measures to strengthen the global monitoring of the TPA epidemic.

Taken together, genomic SNVs and *tpr-*gene-based phylogenetic and phylogeographical analyses not only revealed the emergence of Chinese TPA strains as a new lineage but also clearly delineated the origin and evolutionary process of TPA subspecies during global transmission. However, there are some limitations in this study: 1) the absence of early TPA genome sequences in the phylogeographical analysis might lead to an overly wide range being estimated for the origin of TPA strains and lineages; 2) the absence of European-derived TPA genome sequences might make it difficult to determine the origin of the European TPA epidemic; 3) the absence of Chinese TPA isolates obtained before the 1960s may be an obstacle to tracing the exact ancestor of Chinese TPA strains; and 4) due to the high cost of NGS and of the isolation of TPA strains, only eight genomes of Chinese TPA isolates were investigated here, and their representativeness of common Chinese TPA strains must be studied further in the future.

## MATERIALS AND METHODS

### Sample collection

In this study, we collected *T. pallidum* from the skin lesions of eight patients suffering from early syphilis according to diagnostic criteria [55], including one primary syphilis patient (SHC-0), three secondary syphilis patients with central nervous system (CNS) involvement (SHD-R, SHE-V, Q3) and four secondary syphilis patients (SHG-I2, B3, C3, and K3) (Supplementary Table 1). Written informed consent was obtained from all of the patients. The protocol for investigating human and animal tissues was reviewed and approved by the Ethics Committee of the Shanghai Skin Disease Hospital. New Zealand white rabbits were used for the isolation and propagation of the TPA strain. Animal care was provided in accordance with the procedures outlined in the Guide for the Care and Use of Laboratory Animals under the protocols approved by the Animal Care and Use Committee of the Shanghai Skin Disease Hospital.

### TPA strain isolation and harvest

The isolation and propagation from patients of TPA strains followed a previously described protocol [56]. Briefly, male seronegative New Zealand white rabbits were infected via injected perianal condyloma or chancre exudate into each testicle. During infection, rabbit serum was collected and used for *Treponema pallidum* particle assay (TPPA) and rapid plasma regain test (RPR) every two days. After the development of a firm orchitis, positive TPPA and positive RPR within 30-40 days, the animals were euthanized via the intracardiac injection of sodium phenobarbital, and the testes were removed aseptically. To harvest TPA, the rabbit testicular tissues were dissected out and squeezed several times in heat-inactivated normal rabbit serum (NRS). After being centrifuged at 1,000 x *g* for 10 min, supernatant containing treponemal cells was carefully collected and centrifuged again at 14,100 x *g* for 3 min. The resulting pellet containing treponemal cells was washed twice in PBS buffer and centrifuged at 14,100 x *g* for 3 min. The resulting treponemal suspension was aliquoted into 10 vials, (1 mL in each vial), subsequently confirmed by dark-field microscopy and stored in liquid nitrogen for further use. All of the sequencing samples were from the first passage in rabbits.

### DNA extraction

The bacterial DNA was then extracted from a frozen treponemal suspension using the QIAGEN Genomic-tip 100/G kit (Qiagen Inc., Chatsworth, CA) according to the manufacturer's instructions, and the DNA samples were stored at −20°C until use.

### Library construction and next-generation sequencing

DNA libraries were constructed with genomic DNA extracted using the CTAB method (Illumina, CA, USA) according to the manufacturer's instructions. PE (paired-end) libraries with an insert size of 300 bp were prepared for each sample. The methods of DNA manipulation, including the generation of single-molecule arrays, cluster growth and paired-end sequencing, were performed using a HiSeq 2500 sequencer (Illumina, CA, USA) with the default parameters according to standard protocols. The Illumina base-calling pipeline was employed to process raw fluorescent images and call sequences. Raw reads of low quality obtained from paired-end sequencing (those with consecutive bases covered by fewer than five reads) were discarded. All of the sequence data (fastq) were deposited in the NCBI SRA database under project number PRJNA305961.

### Data analysis and SNV annotation

Sequence reads were purified using the FASTX toolkit (http://hannonlab.cshl.edu/fastx_toolkit/). After adaptor trimming and quality trimming, the clean reads were mapped to the Nichols reference genome (GenBank accession: CP004010.2) using Bowtie2 with the very-fast-local parameter. The parameter values were as follows: max 0 mismatch and 0 gap in a seed alignment, excluding the reads after 5 failed attempts to extend a sequence. Other parameters were set as default parameters. SNVs from the alignments were called using SAMTools [57] and output in pileup format. To validate the resulting non-redundant candidate SNVs in eight sequenced genomes, the numbers of the most abundant (n1) and second most abundant (n2) nucleotides at each SNV in each strain (according to the number of reads in each strain supporting the presence of the nucleotide) were examined. High-quality SNVs satisfied the following criteria: (i) the most abundant base was different from that in the Nichols genome; (ii) n1 + n2 ≥ 10; and (iii) n1/(n1+n2) ≥ 0.7.

### Phylogenetic and phylogeographical analyses of treponemal strains based on genomic SNVs and *tpr* genes

To investigate the genetic diversity of Chinese TPA strains, a total of 329 SNVs in the Nichols strain were collected and used for phylogenetic analysis. Phylogenetic trees of genomic SNVs were reconstructed through a maximum likelihood (ML) heuristic search in phylogenetic analysis using parsimony (PAUP) v4.0 b10, and the general time-reversible model with a proportion of invariant sites and a gamma distribution (GTR+Ɣ+R) was selected as the most appropriate analysis model using ModelTest software [58]. To generate the maximum-likelihood tree, the statistical robustness of the tree and the reliability of the branching patterns were confirmed through bootstrapping tests for 1,000 replicates, and the effective phylogenetic relationship was supported by a >80% bootstrap value. To confirm the genetic linkage as indicated by the ML tree, a Bayesian inferred tree was also reconstructed using MrBayes 3.1 as previously described [34]. To fully elucidate the origin of Chinese-derived TPA strains, additional phylogenetic analyses of both the *tpr* genes and genomic SNVs were performed using PAUP 4.0 software, and phylogeographical analyses were conducted using the BEAST v1.6.2 software package according to the manufacturer's instructions [59]. Each MCMC analysis was run for at least 50 million generations and sampled every 10,000 generations in BEAST V1.6.2. The program Tracer V1.5.1 (tree.bio.ed.ac.uk/software/tracer/) was employed to determine convergence and to determine whether the effective sample size (ESS) was >200. When the ESS was <200, the MCMC chain length was elongated to 100 million. To construct maximum clade credibility (MCC) trees, the initial 25% of trees generated were discarded as burn-in, and the living trees per run were summarized using Tree Annotator, as implemented in BEAST V1.6.2. All of the trees were assessed and edited using FigTree V1.3.1 (tree.bio.ed.ac.uk/software/figtree/), which was also employed to estimate the time of the most recent common ancestor (tMRCA) of various nodes on the MCC tree. Posterior probabilities for the internal nodes were calculated from the posterior density of the trees. The statistical uncertainty in parameter estimates was reflected based on the values of the 95% highest posterior density (HPD) credible region (CR).

### SIFT analysis and three-dimensional (3D) protein-folding structure prediction

The SIFT algorithm was applied to predict SNVs that are likely to affect functions of penicillin regulatory protein. Briefly, SIFT identifies homologs of the gene of interest in other bacteria by 1) scoring the conservation of the positions where mutations are detected and 2) weighting this score according to the nature of the amino acid change. These measures are incorporated into a normalized probability score, and a SIFT score ≤0.05 indicates a predicted functional impact. The recommended >3.5 conservation threshold was used to filter biased predictions. All of the mutated penicillin regulatory genes found in TPA strains were used for SIFT analysis. Predictions of SNVs that destabilize the protein structure were obtained using the Protein Homology/analog Y Recognition Engine V 2.0 [60].

### PCR-based Sanger sequencing

We selected 23 candidate SNV sites and their flanking sequences, including 6 SNVs in the PBP gene, 7 SNVs in the *tpr* gene and 10 SNVs in other genes. The PCR primers were designed using Primer 5.0 software to fall within 100 nt upstream or downstream of the position of SNV and were used to amplify these regions by PCR. The PCR product preparation and Sanger sequencing were performed by Life Technologies. Sanger sequencing was performed using the BigDye Terminator v1.1 Cycle Sequencing Kit. After ethanol purification, the PCR products were run on an ABI 3730 sequencer. The resulting data were screened for mutations through Bio-Edit software alignment.

## SUPPLEMENTARY MATERIAL










