# Supplementary material for: Tracing the origin of Treponema pallidum in China using next-generation sequencing
Source: Oncotarget. 2016 Jun 17;7(28):42904–18. doi: 10.18632/oncotarget.10154 (PMC5189996; doi:10.18632/oncotarget.10154)
Supplement: Supplementary file 1 [file oncotarget-07-42904-s001.pdf]

Tracing the origin of *Treponema pallidum* in China using next-generation sequencing

Supplementary Material

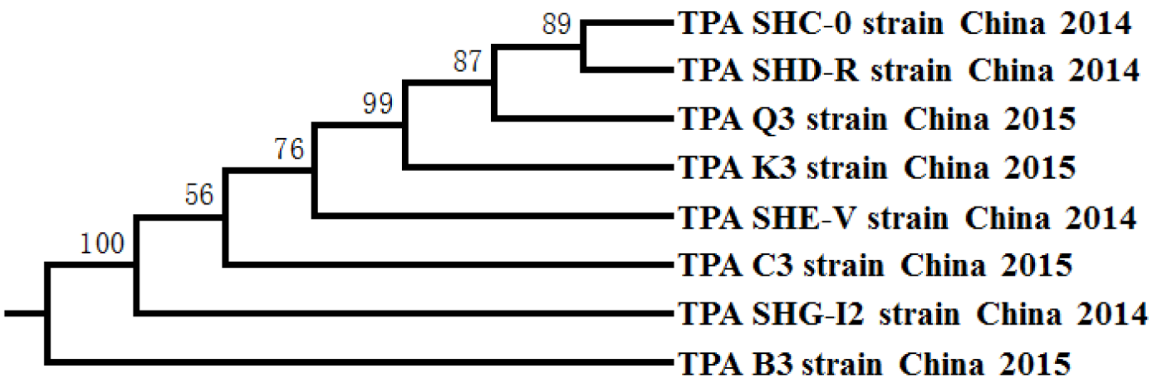

Supplementary Figure 1: The ML tree of Chinese TPA strains. The displayed phylogenetic tree was formed by eight Chinese TPA isolates based on their genomic SNVs.

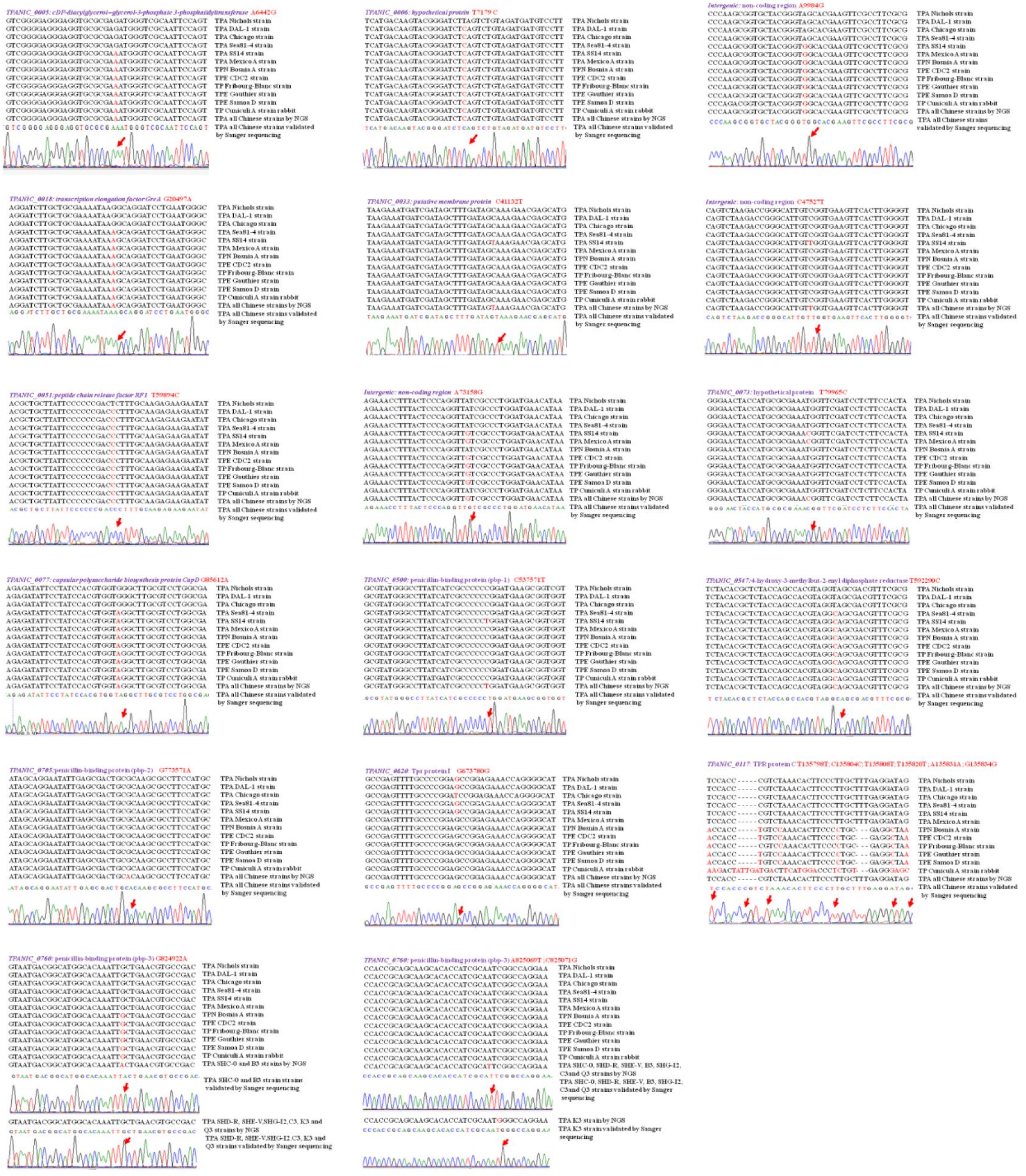

Supplementary Figure 2: SNVs as validated by Sanger sequencing. A total of 23 SNVs that were identified through NGS were validated by PCR-based Sanger sequencing.

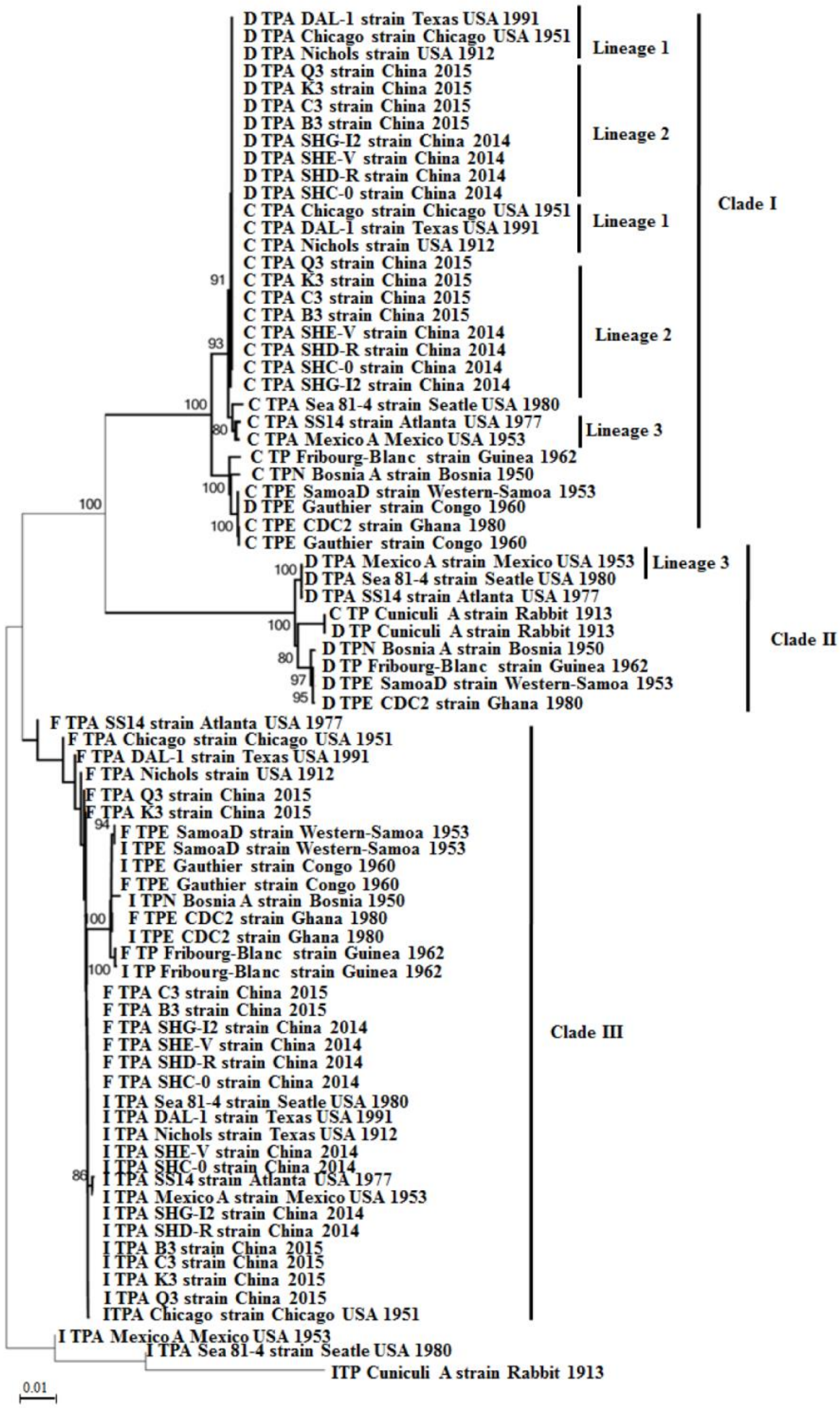

Supplementary Figure 3: Phylogenetic analysis based on *tpr* Subfamily I genes of *T. pallidum*. Three distinct clusters supported by a greater than 99% bootstrap probability were identified. Clade I included all of the *tpr*C genes and some of the *tpr*D genes (mainly from TPA Lineages 1 and 2); Clade II included the *tpr*D genes from the TPA strains (SS14, Mexico A and Sea81-4) and TPE strains; and Clade III included the *tpr*F and *tpr*E genes.

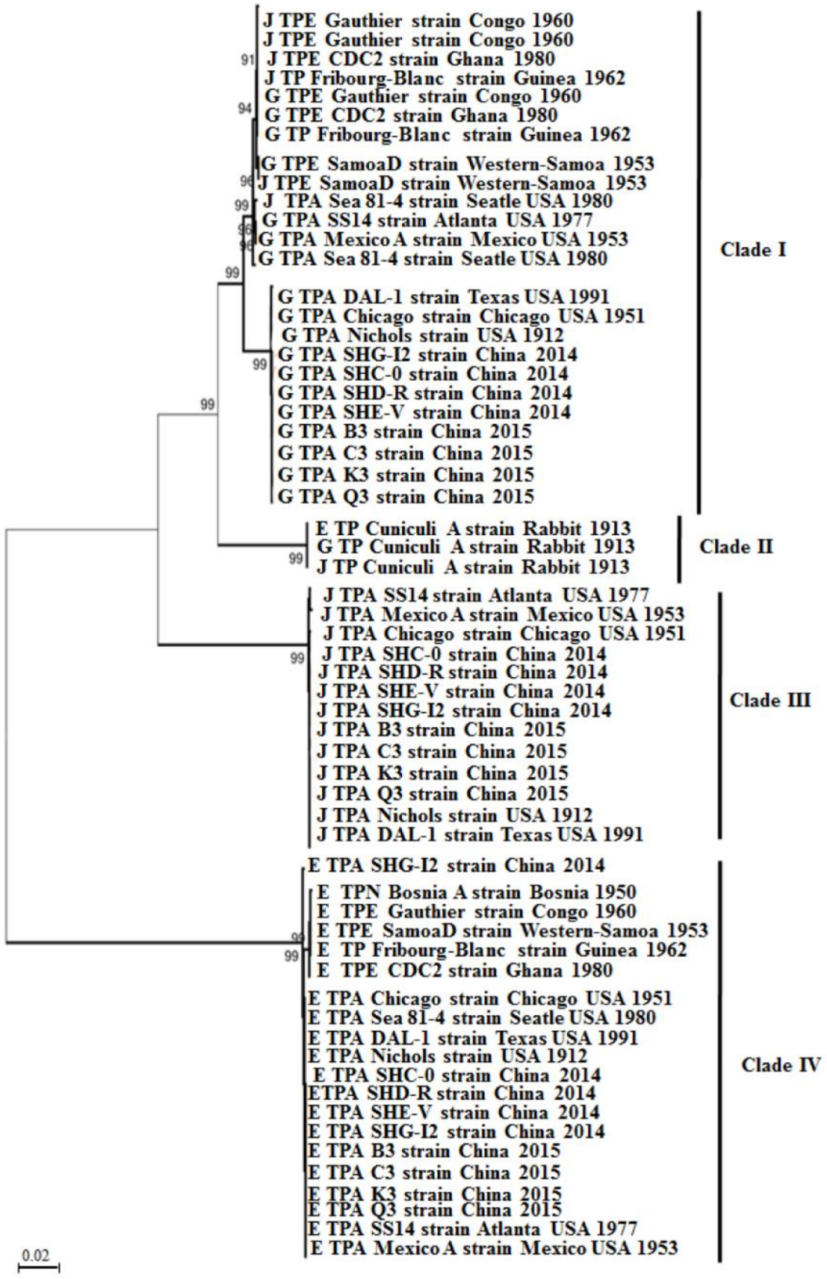

Supplementary Figure 4: Phylogenetic analysis based on *tpr* Subfamily II genes of *T. pallidum*. Four distinct clusters supported by a greater than 90% bootstrap probability were identified. All of the *tpr*G genes and TPE and TPN-derived *tpr*J genes formed Cluster I; *T. paraluiscuniculi* strain-derived *tpr*E, *tpr*G and *tpr*J genes formed Cluster II; all of the TPA-derived *tpr*J genes formed independent Cluster III; and all of the *tpr*E genes derived from treponemes (except for the *T. paraluiscuniculi* strain) formed Cluster IV.

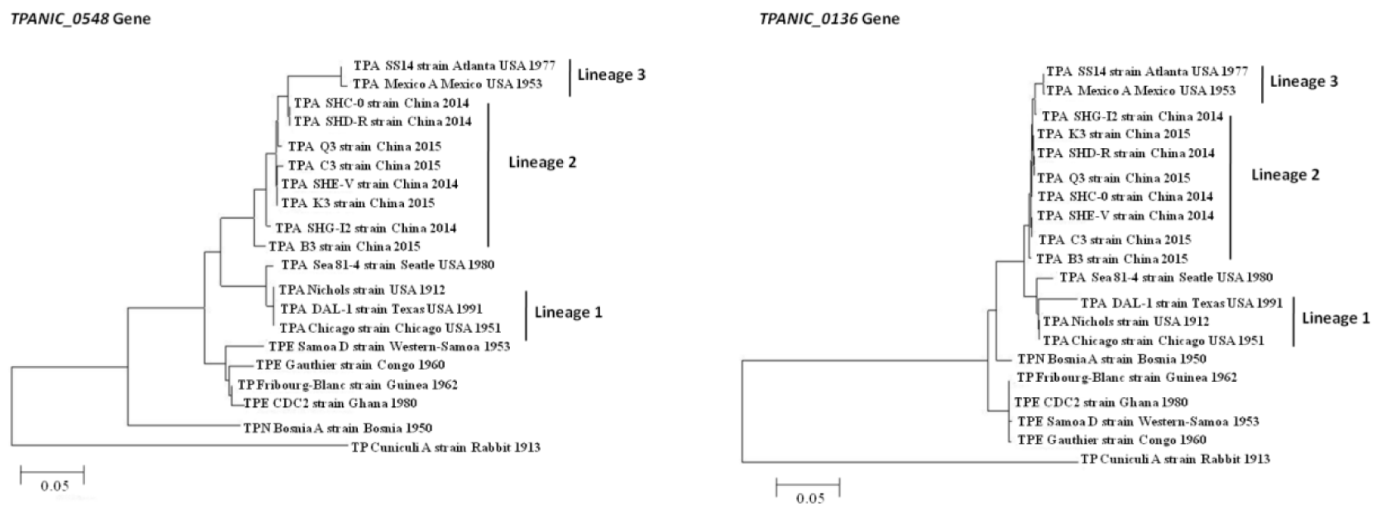

Supplementary Figure 5: The ML tree of the *TPANIC_0548* gene and *TPANIC_0136* gene. Two distinct clusters supported by a greater than 90% bootstrap probability were identified in 14 TPA strains. One cluster consisted of TPA Lineages 2 and 3, and another was formed by TPA Lineage 1.
